# Aluminosilicate Nanocomposite on Genosensor: A Prospective Voltammetry Platform for Epidermal Growth Factor Receptor Mutant Analysis in Non-small Cell Lung Cancer

**DOI:** 10.1038/s41598-019-53573-9

**Published:** 2019-11-19

**Authors:** Santheraleka Ramanathan, Subash C. B. Gopinath, M. K. Md Arshad, Prabakaran Poopalan, Periasamy Anbu, Thangavel Lakshmipriya, Farizul Hafiz Kasim

**Affiliations:** 1 Institute of Nano Electronic Engineering, Universiti Malaysia Perlis, 01000 Kangar, Perlis Malaysia; 2 School of Bioprocess Engineering, Universiti Malaysia Perlis, 02600 Arau, Perlis Malaysia; 3 School of Microelectronic Engineering, Universiti Malaysia Perlis, Pauh Putra, 02600 Arau, Perlis Malaysia; 4 Department of Biological Engineering, College of Engineering, Inha University, Incheon, 402-751 Republic of Korea; 5 Centre of Excellence for Biomass Utilization, School of Bioprocess Engineering, Universiti Malaysia Perlis, 02600 Arau, Perlis Malaysia

**Keywords:** DNA, Diagnostic markers

## Abstract

Lung cancer is one of the most serious threats to human where 85% of lethal death caused by non-small cell lung cancer (NSCLC) induced by epidermal growth factor receptor (EGFR) mutation. The present research focuses in the development of efficient and effortless EGFR mutant detection strategy through high-performance and sensitive genosensor. The current amplified through 250 µm sized fingers between 100 µm aluminium electrodes indicates the voltammetry signal generated by means of the mutant DNA sequence hybridization. To enhance the DNA immobilization and hybridization, ∼25 nm sized aluminosilicate nanocomposite synthesized from the disposed joss fly ash was deposited on the gaps between aluminium electrodes. The probe, mutant (complementary), and wild (single-base pair mismatch) targets were designed precisely from the genomic sequences denote the detection of EGFR mutation. Fourier-transform Infrared Spectroscopy analysis was performed at every step of surface functionalization evidences the relevant chemical bonding of biomolecules on the genosensor as duplex DNA with peak response at 1150 cm^−1^ to 1650 cm^−1^. Genosensor depicts a sensitive EGFR mutation as it is able to detect apparently at 100 aM mutant against 1 µM DNA probe. The insignificant voltammetry signal generated with wild type strand emphasizes the specificity of genosensor in the detection of single base pair mismatch. The inefficiency of genosensor in detecting EGFR mutation in the absence of aluminosilicate nanocomposite implies the insensitivity of genosensing DNA hybridization and accentuates the significance of aluminosilicate. Based on the slope of the calibration curve, the attained sensitivity of aluminosilicate modified genosensor was 3.02E-4 A M^−1^. The detection limit of genosensor computed based on 3σ calculation, relative to the change of current proportional to the logarithm of mutant concentration is at 100 aM.

## Introduction

Cancer is an endangering health disease with the limited therapeutics to date, and ends human life to death. Among all, lung cancer stands in the first place, causing the highest number of deaths a year. Statistics released by International Agency for Research on Cancer reports that 2.09 million new cases were diagnosed in 2018 and 18.4% of the cases lead to cancer deaths, estimating ~1.7 million lung cancer deaths a year^1–3^. In a year, about 85% of the lung cancer patients reported are diagnosed with non-small cell lung cancer (NSCLC), known as adenocarcinoma where the rest are diagnosed with a small lung cancer cell. NSCLC recognized on the basis of epidermal growth factor receptor (EGFR) mutation, act as the prophetic constituent contributes to the lethal death^4–6^. Deletion in exon 19 and a single point mutation in exon 21 prompt the generation of EGFR tyrosine kinase inhibitor, results in exhaustive NSCLC cell proliferation, being the precursor for the spread of lung cancer cells in human. Intended therapeutics and prognosis monitoring for lung cancer screening in the early stage of the diagnosis relies on the detection of EGFR mutation^7–10^. On such a premise, efficient and effortless detection strategies of EGFR mutation are mandatory. Emerged detection of EGFR mutation through direct sequencing, high performance liquid chromatography, pyrosequencing and polymerase chain reaction (PCR) analysis hasn’t met the effective and ideal strategy due the highly expensive experimental procedures, which involves the experts to handle it and time consuming procedures^11–14^. Some techniques based on optical and fluorescence, voltammetry, surface-enhanced roman spectroscopy (SERS) and surface plasmon resonance (SPR) have been developed eventually for quick and effective EGFR mutation detection^11,15–17^. Although various great works have been accomplished, advanced devices and procedures with high sensitivity for early NSCLC detection are highly desired.

In the last decade, the application of biosensors in the detection of blood biomarkers, and proteins have received high attention in the diagnosis of various diseases^18,19^. Apart from the lab-based equipment, biosensors with inexpensive inventing technology and low consumption of clinical samples are highly welcomed as the easier clinical diagnostic tool^20,21^. Presently, there is a huge interest in the invention of genosensor for the detection of highly sensitive DNA. Genosensor is expected to be widely applicable in pathogenic diseases detection, clinical diagnosis, and forensics involving genomic DNA sequences. It has defined as a suitable detection device for the genomic DNA associated diseases. Lung cancer is one of the endangered gene associated disease where high sensitive genosensor is desired to detect a single point EGFR mutation. Voltammetry detection has been recognized as one of the best in the detection of DNA hybridization where the charge carried by the genomic biomarkers was literally converted into a readable signal. Voltammetry genosensor is preferred in contrast with other sensing methods due to its quick response, minimal effort in the invention with low cost and less time consuming^22,23^. The two key challenges in the invention of genosensor are the detection limit and its enhanced sensitivity by using the least amount of DNA samples for immobilization on the sensing surface. For the excellent hybridization and immobilization of efficiency, nanomaterials have studied for the surface modification of genosensor. In recent decade, gold, silver and titanium oxide nanoparticle are applied on the sensing surface to increase the surface area for DNA immobilization and hybridization due to its physiochemical property, developing a significant enhancement for genosensor sensitivity^17,24–28^.

In this research, we have presented a new genosensor surface functionalization using aluminosilicate nanocomposite extracted from the joss fly ash for early detection of EGFR mutation in NSCLC. The extremely burned and wasted joss fly ash was significantly used in the research to synthesize aluminosilicate nanocomposite, an excellent nanomaterial for enhancing voltammetry genosensor sensing. The porous crystalline aluminosilicate is well known for its effective cation exchange property and as an electrochemical catalyst in various biomedical applications^29–34^. A voltammetry interdigitated electrode (IDE) was designed as it is highly precise for the genomic DNA hybridization with microscale aluminium electrodes and gaps. A series of surface chemistry were conducted on the sensing surface to ensure the strong binding of aluminosilicate nanocomposite on the genosensing surface. To detect the EGFR mutation using the aluminosilicate nanocomposite modified genosensor, genomic DNA probe is designed as it is complementary for the genomic sequence (mutant type), indicates the presence of EGFR mutation. Whilst, the wild-type genomic sequence with one base pair mismatch indicates the absence of EGFR mutation yet it denotes the sensitivity of genosensor in the detection of EGFR mutation with single base pair mismatch. The sensitivity of genosensor was evaluated based on the signal amplified from the hybridization of genomic DNA sequencing using picoammeter/voltage source. The non-complementary single stranded DNA (ssDNA) designed as control genomic sequence for the specificity evaluation of genosensor. With all of the above, the research motivates on the development of efficient and effortless strategy in detecting EGFR mutant through quick voltammetry response, in conjunction with low cost intervention of aluminosilicate nanocomposite from joss fly ash.

## Experimental

Aluminosilicate nanocomposites extracted from joss fly ash collected in a local Chinese temple in the Northern region of Malaysia. 1,1′-Carbonyldiimidazole (CDI), Tween-20 detergent, Tris-buffer, (3-Aminopropyl)triethoxysilane (APTES), phosphate-buffered saline (PBS), 1-Ethyl-3-(3-dimethylaminopropyl)-carbodiimide (EDC), and N-hydroxysuccinimide (NHS) used for the surface enhancement of genosensor were procured from Sigma Aldrich, USA. Genomic sequences designed to detect EGFR mutation were purchased from Integrated DNA Technologies, USA. Carboxyl-terminated genome (5′ COOH-C6-CAGCAGTTTGGCCCGCCCAAAA 3′) was used as a probe for DNA immobilization. The complementary mutant strand (5′ TTTTGGGCGGGCCAAACTGCTG 3′), single base pair mismatch strand (5′ TTTTGGGCTGGCCAAACTGCTG 3′) and non-complementary strand (5′ CAGCAGTTTGGCCCGCCCAAAA 3′) were used as targets in the detection strategies^20^.

### Development of aluminosilicate nanocomposite modified genosensor

Aluminosilicate nanocomposite was synthesized from the extremely found joss fly ash through simple experimental steps. Initially, the fly ash was treated with 10% sulfuric acid (H_2_SO_4_) and the ash was separated from the solution by centrifugation. The solution was titrated until pH 7 and the gel formed was washed and purified for characterization. Then, the acid treated ash was proceeding for alkaline treatment with 2.5 M of NaOH solution. The solution was separated from the ash and titrated until neutral pH. The gel formed at pH 7 indicates the presence of aluminosilicate nanocomposite. Aluminosilicate nanocomposite extracted from joss fly ash were analysed by electron characterization, field emission scanning electron microscope (FESEM, Hitachi, S-4300 SE, Japan) and field emission transmission electron microscope (FETEM, JEM-2100F, JEOL, Japan) and Atomic force microscopy (AFM; Nanoscope, Ica, Veeco, USA) to visualize the shape and size of nanoparticle and ensure the yield of high purity aluminosilicate nanocomposite from the joss fly ash. Conventional photolithography technique was applied in the development of the voltammetry sensing device. The substrate was undergone a wet thermal oxidation to form an active oxide layer on the surface. To enhance the current conductivity of genosensor, a thin layer of aluminium was deposited on the oxide surface. The positive photoresist (PR) was coated on the SiO_2_ substrate deposited with aluminium were allowed to undergone UV light exposure for the IDE pattern transfer on the sample surface from the designed chrome mask. The transferred pattern was developed. The fabricated IDE was examined under high power microscope (HPM), 3D Profilometer (Hawk 3D nano-profiler), and FESEM to justify the development of genosensor with high accuracy in dimension and denotes simple, low-cost and time saving conventional photolithographic technique.

### Surface enhancement of genosensor

**Figure 1:**
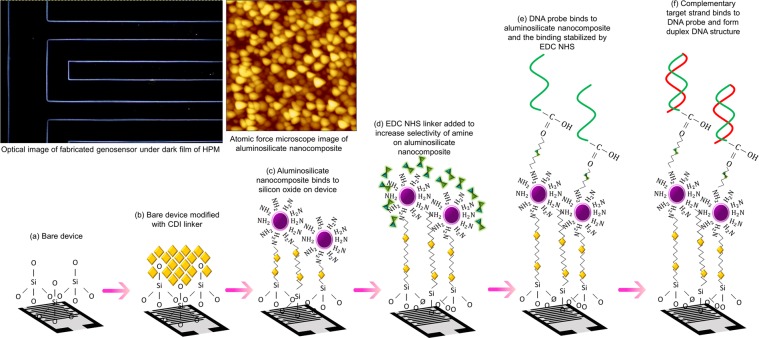
Schematic illustration of surface functionalization. Conducted on bare silicon substrate was from deposition of aluminosilicate nanocomposites modified with APTES to immobilization of DNA probe and targets, generating high performance genosensor. Figure insets are displaying the surface morphology.

A series of chemical modification on the fabricated IDE is necessary to ensure the proper immobilization of DNA strands on the nanomaterial modified sensing surface. Figure 1 illustrates the modification performed on the IDE surface to generate genosensor for DNA mapping and immobilization. A 100 mg of aluminosilicate nanocomposite extracted from joss fly ash was immersed in 2% APTES solution for 1 hour, enables the formation of amine groups. CDI solution with 0.5 M concentration was dropped on the sensing surface and incubated for 1 hour at room temperature. Then, 10 µL of aluminosilicate modified with APTES solution was added on the IDE sensing surface, allowed to react for 1 hour. CDI enables the chemical bonding between SiO_2_ on the silicon substrate and the amine on the surface of aluminosilicate nanocomposite. Then, IDE sensing surface was washed with distilled water and 0.05% Tween 20 solution to remove unbound nanoparticles and to block the unreacted SiO_2_ on a silicon substrate. An equal amount of EDC and NHS solutions were mixed and 10 µL of the solution was dropped on the sensing surface. A 1 µM of DNA probe designed with a carboxyl group at the 5′ end of the strand, diluted with PBS buffer were immobilized on the sensing surface for 15 minutes. EDC is a carbodiimide crosslinker responsible in activating the carboxyl groups for spontaneous reaction with primary amines, whereas NHS is responsible in converting carboxyl groups to amine-reactive NHS esters^35,36^. Thus, the carboxyl group on the DNA probe binds with the amine group on aluminosilicate nanocomposite through a series of chemical modifications aided by EDC and NHS linkers. The sensing surface ensured to be wet using PBS buffer, after immobilizing the DNA probe to make sure the secondary structure of DNA was undisrupted. 0.05% Tween 20 solution acts as a blocking agent. It blocks the unwanted sample surface and enables the ssDNA probe were easily available for DNA hybridization. Then, the target DNA strand was added on the sensing surface forming DNA hybridization with the probe depending on its matching genomic sequence. Fourier Transform Infrared (FTIR, Spectrum 65, Perkin Elmer, USA) spectroscopy was performed for genosensor at the interval of every step on the modification with surface chemistry to evaluate the chemical and structural bonding on the sensor for high-density DNA immobilization.

### Genosensing detection strategy

The voltammetry signals generated by DNA hybridization between probe and target sequences were interpreted using a quantitative electrical measurement. Keithley 6487 picoammeter were used to investigate the electrical characterization of genosensor modified with aluminosilicate nanocomposite in conjunction with the EGFR mutation detection based on DNA hybridization. The 2-point probe was connected to the terminal voltages, which were set from 0 V to 2 V and measured the current generated. For the detection of EGFR mutation, different concentrations of mutant type target where prepared using PBS buffer. The concentration was diluted from 100 nM to 1 aM range. After immobilizing 1 µM of DNA probe followed by blocking and washing steps, 10 µL of 1 aM target concentration was dropped on the sensing surface of genosensor and incubated for 10 minutes. After the incubation period, the surface was rinsed with 10 µL PBS buffer before measuring the electrical signal using picoammeter. The similar steps were repeated for all the concentrations and with one base mismatch genomic target (wild type) and non-complementary sequences. To validate the efficiency of aluminosilicate nanocomposite modified genosensor, the detection of EGFR mutation was examined using genosensor without nanomaterial surface enhancement.

## Results and Discussion

**Figure 2:**
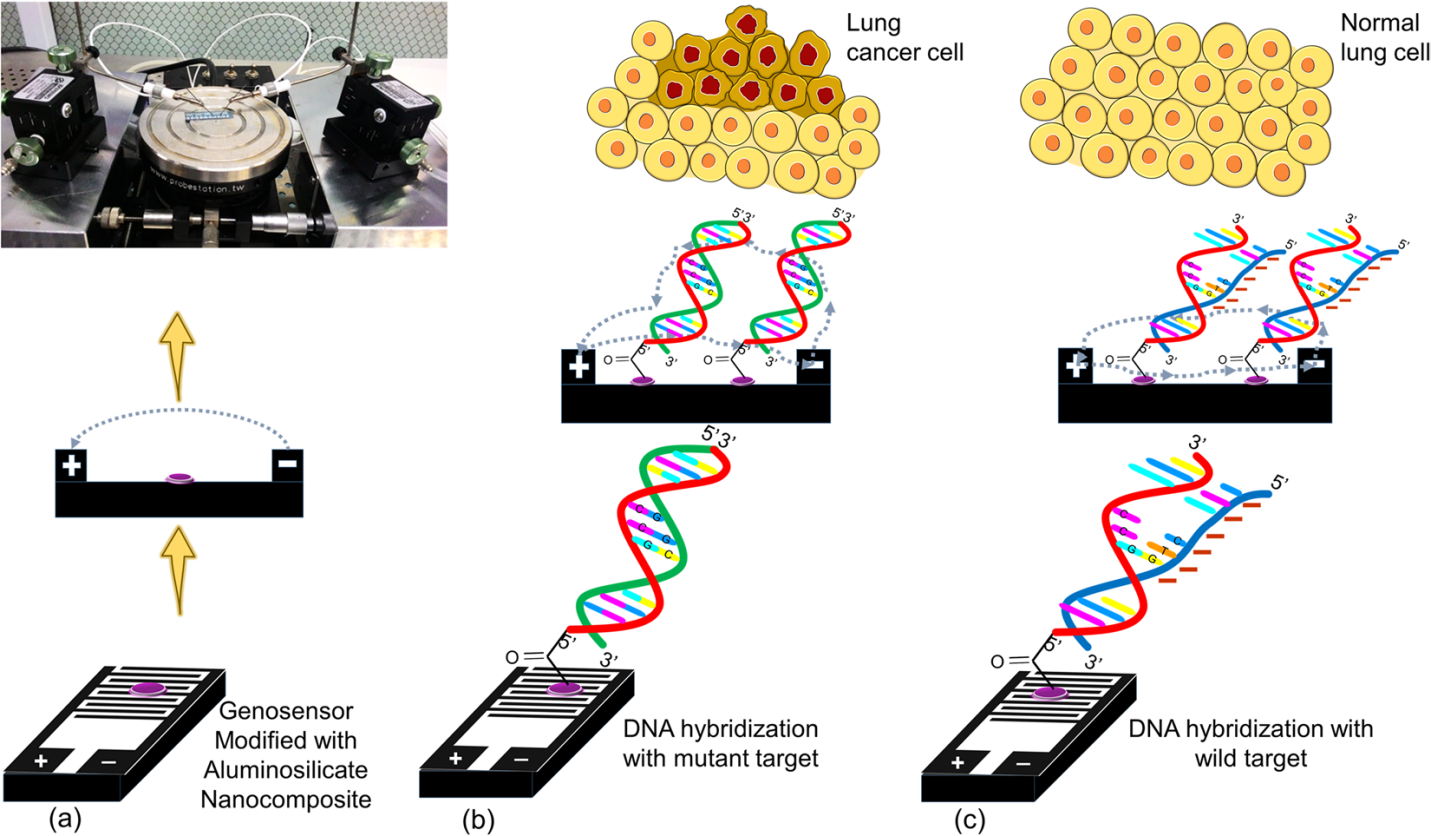
Detection strategy of EGFR mutation using aluminosilicate modified genosensor. (**a**) Detection of the voltammetry signal amplified from bare genosensor, measured by Picoammeter. (**b**) Voltammetry signal amplified through genosensor induced from the complementary DNA hybridization between the probe and mutant strand, indicating EGFR mutation. (**c**) Voltammetry signal illustrates the sensitivity of genosensor in detecting hybridization between the probe and one base mismatch wild type referring to the absence of EGFR mutation.

Figure 2 demonstrates the detection strategy of EGFR mutation using genosensor. Aluminosilicate nanocomposite improves the electrical conductivity of genosensor, due to not only the large surface area/volume, but also its excellent cation exchange property which enhances the flow of charge carried through the electrodes. The sensitivity in hybridization of genomic DNA strands designed for the detection of EGFR mutation indicated by the current conducted through the electrodes of the genosensor. The genosensor assembled in the present research was validated using complementary mutant DNA strand and one base mismatch DNA strand in the hybridization against DNA probe indicating the occurrence of NSCLC via the voltammetry signal amplified through the genosensor.

### Characterization of aluminosilicate nanocomposite modified genosensor

**Figure 3:**
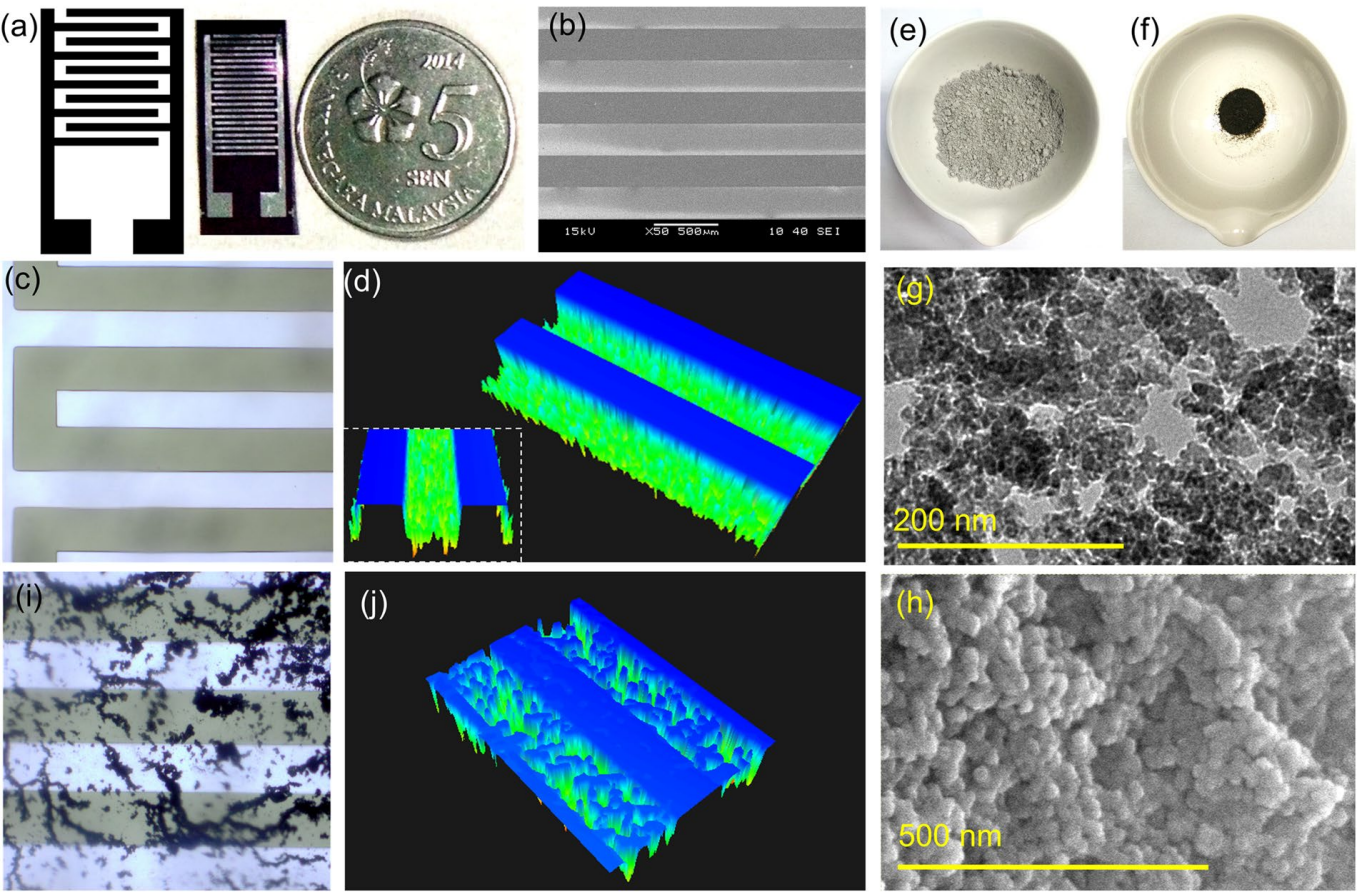
(**a**) Design and characterization of genosensor. The basic design and the generated genosensor are shown on the left. (**b**) Image of genosensor captured under a scanning electron microscope at 500 µM, showing the fingers of the aluminium electrode and the gaps between the electrodes. (**c**) HPM image of fabricated genosensor. (**d**) 3D image of fabricated genosensor before depositing aluminosilicate nanocomposites (inset figure shows front view) using 3D Profilometer. (**e**) Joss fly ash used to synthesize aluminosilicate nanocomposites. (**f**) Aluminosilicate extracted from joss fly ash (**g**) FETEM image of the nanocomposites. (**h**) Uniformly dispersed spherical shape of aluminosilicate nanocomposites under FESEM analysis. (**i**) Image of genosensor under HPM after deposition of nanocomposites. (**j**) 3D image of aluminosilicate nanocomposite modified genosensor illustrates the nanocomposites only attach on the SiO_2_ gap without disrupting the aluminium electrodes.

Figure 3a shows the basic design of IDE and the generated genosensor in the present research. The overall dimension of genosensor fabricated was 6 mm by 15 mm. The width of aluminium fingers was 100 µm and the gap between the fingers was 250 µm. The precision of the fabricated genosensor was affirmed by FESEM (Fig. 3b), and HPM (Fig. 3c) analysis. The dark film optical image of IDE under HPM analysis is shown in Fig. 1 (inset). 3D dimension image of genosensor was visualized under 3D nanoprofilometer as shown in Fig. 3d. The height of the aluminium electrode examined was ~26 µm. The characterization was performed on the fabricated genosensor promises the successful conventional photolithography technique in generating the voltammetry genosensor. Aluminosilicate nanocomposite was successfully extracted from the joss fly ash (Fig. 3e). Figure 3f shows the nanomaterial synthesized from the joss fly ash waste, where the gel formed under an alkaline condition at neutral pH dried and obtained in powder form. Figure S1 shows the experimental samples in the process of synthesizing aluminosilicate nanocomposite. Electron microscopic analysis of aluminosilicate nanocomposite revealed image of nanocomposite obtained under FETEM (Fig. 3g). FESEM reveals the uniform particle distribution with the spherical shape at 500 nm magnification as shown in Fig. 3h, also implies the size of the particle obtained were ∼25 nm. In addition, the uniform distribution of sharp edged aluminoslicates under atomic force microscope is shown in Fig. 1 inset. The energy disperses spectroscopy obtained under FESEM analysis showed the ratio of silica to aluminium was 13.24 to 7.96 (1.66) justifies the purity of aluminosilicate nanocomposite synthesized from the environmental waste substances. To enhance the sensing surface of genosensor, aluminosilicate nanocomposite was deposited on the surface of genosensor, improves the conductivity and sensitivity. To affirm the particle deposition on the sensor, it was characterized under HPM and 3D profilometer. Figure 3i shows the huge amount of particles dispersed on the sensing surface of genosensor, observed under HPM. The image only reflects the presence of nanocomposites on genosensor, without validating the chemical bonding between nanocomposites and genosensing surface. Figure 3j shows the image of genosensor modified with aluminosilicate nanocomposite under 3D nanoprofilometer. The 3D image justifies the attachment of nanomaterial on the silicon oxide layer only without disrupting the conductive aluminium electrode. It validates the proper surface functionalization, which is the deposition of nanocomposites performed on genosensor as the aluminosilicate binds to SiO_2_ layer only. The signals were amplified from the silicon oxide layer and transmitted through the aluminium electrodes from the negative to positive terminal voltages.

### Surface chemistry characterization of genosensor

**Figure 4:**
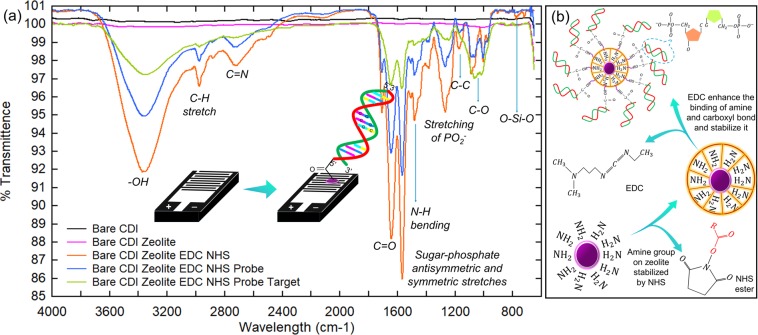
(**a**) FTIR analysis. Conducted on genosensor affirms the surface functionalization upon every step of the modification process. (**b**) Illustration of EDC and NHS interaction as chemical linkers with biomolecules for high binding affinity.

Figure 4 shows the FTIR spectra on genosensor characterized at every step with surface functionalization. Bare genosensor functionalized with CDI linker shows a minor peak formed in the FTIR spectra at 800 cm^−1^ denotes the existence of SiO_2_ on the sensing surface. The addition of aluminosilicate nanocomposite modified with APTES on the sensing surface has shown peak appearance at 1030 cm^−1^ indicates functionalization between carbon chain in APTES and the oxide layer of the sensor, raised due to the bending of C-O bonding. An extreme peak variation was observed in the spectra when EDC and NHS mixed solution was dropped on the genosensor. EDC and NHS possess multiple chemical bonding which functionalizes the existing amine group on the aluminosilicate nanocomposite. Figure 4b shows the chemical structure of NHS ester and EDC and its involvement in the surface chemistry of genosensor^37,38^. When EDC and NHS were added, the peak formed at 1080 cm^−1^ represents the C-C bonding. N-H bending in both EDC and NHS were responsible for the peak appearance at 1270 cm^−1^. The prominent peak observed at 1560 cm^−1^ and 1540 cm^−1^ indicates the strong C = O and N-H deformation in the chemical structure of EDC and NHS when it has reacted with APTES modified aluminosilicate nanocomposite. C = N and C-H stretch were affirmed by the peak formed at 2725 cm^−1^ and 2925 cm^−1^, respectively. The protruding peak observed at 3360 cm^−1^ illustrates the hydroxyl (OH) present on the sensing surface due to the washing steps with water and buffers. When the ssDNA probe was immobilized on the sensing surface, a peak appeared from 1200 cm^−1^ to 1000 cm^−1^ represents the stretching of PO_2_^−^ functional group present on the surface of the nucleotide strand. When the complementary mutant target has immobilized, duplex DNA strand formed between probe and target giving peak response from 1150 cm^−1^ to 1650 cm^−1^ indicates the strong sugar-phosphate backbone of DNA strand. The hydrogen bonds held the gene on DNA strands notable from 3000 cm^−1^ to 3500 cm^−1^ ^39–43^. The intensity of transmittance reduces from EDC and NHS mixture to DNA duplex structure on the sensing area is due to the amendment in the surface chemistry of genosensor where the functional groups present on the sensing surface were reduced and trimmed at every step to ensure the voltammetry signal generated by genosensor is only amplified from the hybridization of DNA strands.

### EGFR mutation detection

The high sensitive genosensor with enhanced sensing surface using aluminosilicate nanocomposite were examined to detect EGFR mutation based on the genomic DNA sequences designed. The ssDNA probe forms DNA duplex with complementary mutant DNA strand indicates the occurrence of EGFR mutation in the presence of NSCLC. ssDNA probe carries a negative charge due to the phosphate group in its structure. The accumulated charge induces the flow of electric current through the aluminium electrode and amplifies the current through Picoammeter. The presences of aluminosilicates on the genosensor surface improve probe immobilization and allow a large density of probe accumulation on the sensing surface. Since the nanomaterial has a high heterogeneous catalyst property, it improves the current amplification through the electrodes of genosensor due to its excellent absorption and ion exchange capacity. When the complementary mutant target was immobilized on the probe attached genosensor, high sensitive DNA hybridization takes place. The DNA duplex structure encloses the negatively charges phosphate functional group and possess sugar-phosphate nitrogenous base. The change in the DNA functional groups creates potential electron shift which flows through the aluminium electrodes from the negative to the positive voltage terminal, generates the voltammetry signal measured by Picoammeter. The accumulation of charge carried on the genosensor is highly influenced by the aluminosilicate nanocomposite which acts as a carrier for the electron movement through the electrodes, improves the conductivity of genosensor and also creates a large surface/volume for biomolecules bonding on the sensing surface, results in the tangible change of current amplified by the sensor.

**Figure 5:**
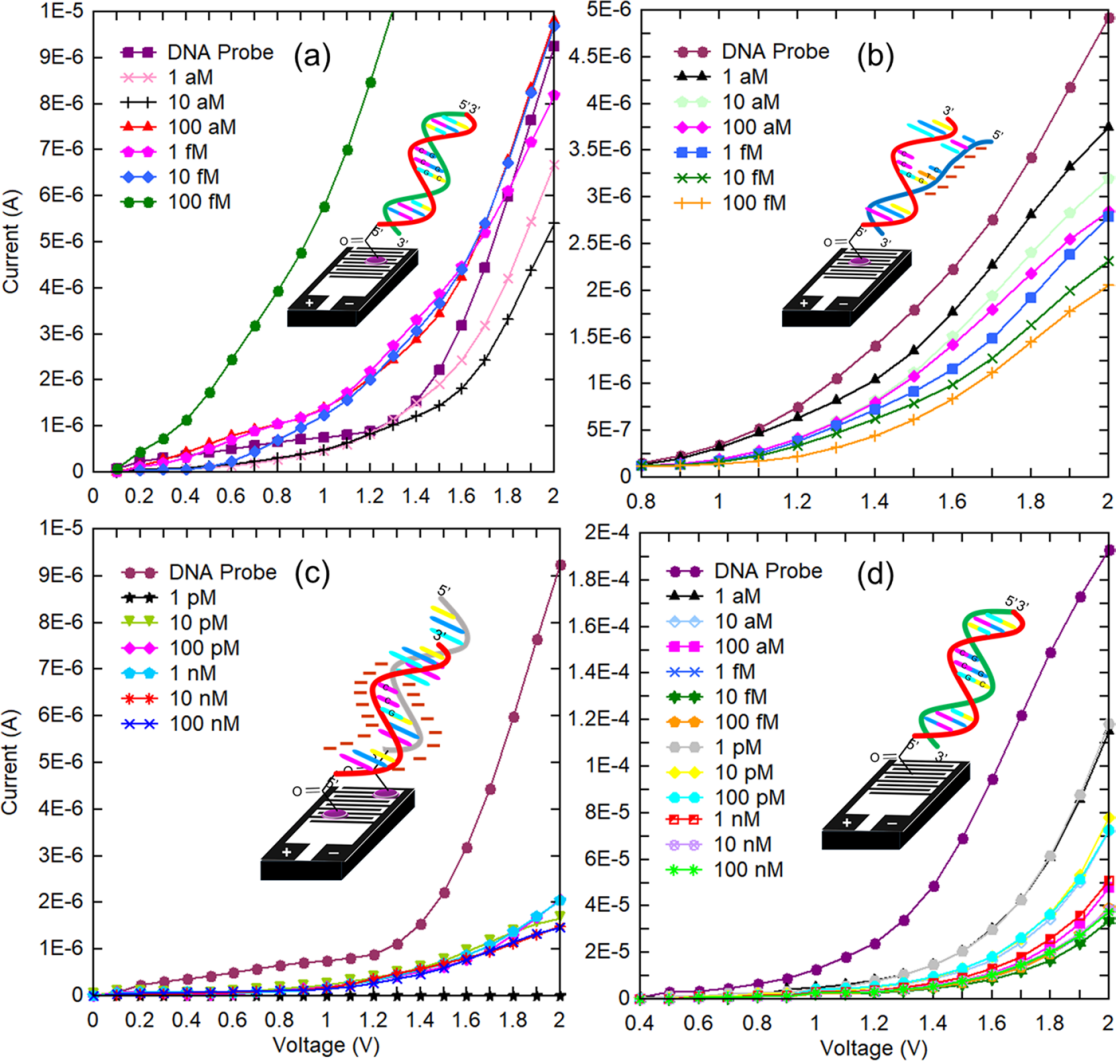
Voltammetry signal amplified by genosensor. DNA probe was allowed hybridizing with (**a**) mutant type, (**b**) wild type and (**c**) non-complementary strand. (**d**) The graph shows current-voltage (I-V) characteristics generated by genosensor without aluminosilicate nanocomposite for surface enhancement when it was investigated against the mutant strand.

In the present research, a series of mutant target concentration were prepared to investigate the detection limit of EGFR mutation using genosensor. Figure 5a shows the current versus voltage graph obtained when the different concentration of mutant target (from attomolar to femtomolar range) was immobilized on the DNA probe attached genosensor. The current amplified at every concentration was examined in contrast to the current conducted by the ssDNA probe. When 1 aM mutant target was immobilized on the genosensor, the curve obtained is below than curve represents probe. This phenomenon indicates the negligible DNA hybridization between probe and target due to the very low concentration of mutant target. The similar pattern is observed for 10 aM target concentration. At 100 aM target concentration, the curve appeared to be above the probe curve indicates the high current amplified by genosensor at 100 aM of the mutant target (Fig. 5a) than probe. The high voltammetry signal amplified by genosensor at 100 aM mutant implies that DNA hybridization took place and the charge accumulated on the sensing surface is large enough to induce the potential electron shift throughout the electrodes and conduct current. It also emphasizes that EGFR mutation is detected at 100 aM mutant concentration against 1 µM probe using genosensor. 1 fM and 10 fM mutant concentrations show a similar trend of the curve as 100 aM mutant, however the current conducted were slightly higher. A distinguished change of current was observed when 100 fM mutant concentration immobilized on the genosensor. The current amplified by genosensor at 100 fM of the mutant is 6 fold higher than the current conducted by 10 fM mutant at 1.5 V indicates the concentration of mutant strands is outrageous for probes to form DNA duplex. At this concentration, the DNA probe has reached its saturation point and it does not available for DNA hybridization. It was proved by the change of current shown at 1 pM of mutant concentration in the Fig. S2, where the curve has fallen below the probe curve and the least amount of current was amplified through the genosensor. The signal generated by the genosensor at 10 pM and 100 pM also show similar trend where the current curves going downwards of the graph as the concentration is increased. It justifies that the genosensor has reached its saturation point and target concentration after 100 fM amplifies irrelevant signal. Based on the results evaluated from Fig. 5a, 100 fM is the excellent concentration for high-density DNA hybridization, however, the lowest detection limit of genosensor was at 100 aM mutant concentration which means the EGFR mutation is able to be detected at 100 aM mutant concentration.

### Specificity of genosensor with one base mismatch and non-complementary genome

The specificity of genosensor was evaluated when the DNA probe attached on aluminosilicate nanocomposite modified genosensor was examined for DNA hybridization with one base mismatch (wild type) genomic sequence. The single base mismatch in the wild type disrupts the formation of DNA duplex with the probe immobilized on the genosensor. Thus, the negatively charged phosphate group on the wild type strand tends to increase the negative charge on the sensing surface. A low electron shift was generated throughout the electrode, results in the weak voltammetry signal. Figure 5b shows the voltammetry signal generated by genosensor when the different concentrations of wild type targets were immobilized. The current generated by genosensor with wild type targets are lower than the current generated by the probe. From 1 aM to 100 fM of wild type, the current reduces gradually and the obtained results were in good agreement with the explanation above where disrupted DNA duplex structure due to single base pair mismatch in the genomic sequence accumulates the negative charge and reduces the electron shift on the genosensor. Hence, the specificity of genosensor was justified by the specificity of one base pair mismatch genomic sequence in the DNA hybridization on the aluminosilicate enhanced sensing surface. This result denotes that genosensor is highly specific in the detection of single base pair mismatch, implies the absence of EGFR mutation. Non-complementary genomic sequence was designed as a controlled genomic sequence to investigate the specificity of genosensor. Figure 5c shows the results obtained when genosensor tested with non-complementary genomic sequence. The voltammetry signal generated by genosensor was much lower compared with the current generated by the probe immobilized on genosensor. Since non-complementary do not bind with the probe, the washing step before measuring the voltammetry signal removes the unbound non-complementary strands from the surface of genosensor, thus the current measured is lower for every concentration as it only depicts the probe attached on genosensor. The performance of genosensor without the surface enhancement using aluminosilicate nanocomposites was evaluated. DNA probes were immobilized on the fabricated IDE using CDI linker, which strengthens the bonding between the silicon oxide layer on sensing surface and DNA probe with a carboxyl group. Mutant type with different concentrations (1 aM to 100 nM) was examined with the genosensor without nanomaterial surface enhancement. Figure 5d shows the results obtained for all the mutant concentration investigated. It shows the voltammetry signal generated by the genosensor at all mutant concentrations is lower than the current generated by the probe, indicating the low sensitivity of genosensor in the DNA hybridization and hence, denotes its inability to detect of EGFR mutation. The absence of aluminosilicate nanocomposite on the genosensor reduces the surface area/volume of the sensing surface for the immobilization of the DNA probe, thus reduces the amount of DNA probes attached on the genosensor. Consequently, least DNA hybridization is expected to take place on the genosensor. The absence of nanomaterial in increasing the surface area/volume, reduces the density of DNA hybridization of the sensing surface, lowers the electron shift throughout the electrodes and hence generates a low voltammetry signal which is unable to detect the mutant target for EGFR detection.

### Voltammetry signal for EGFR mutation: limit of detection

**Figure 6:**
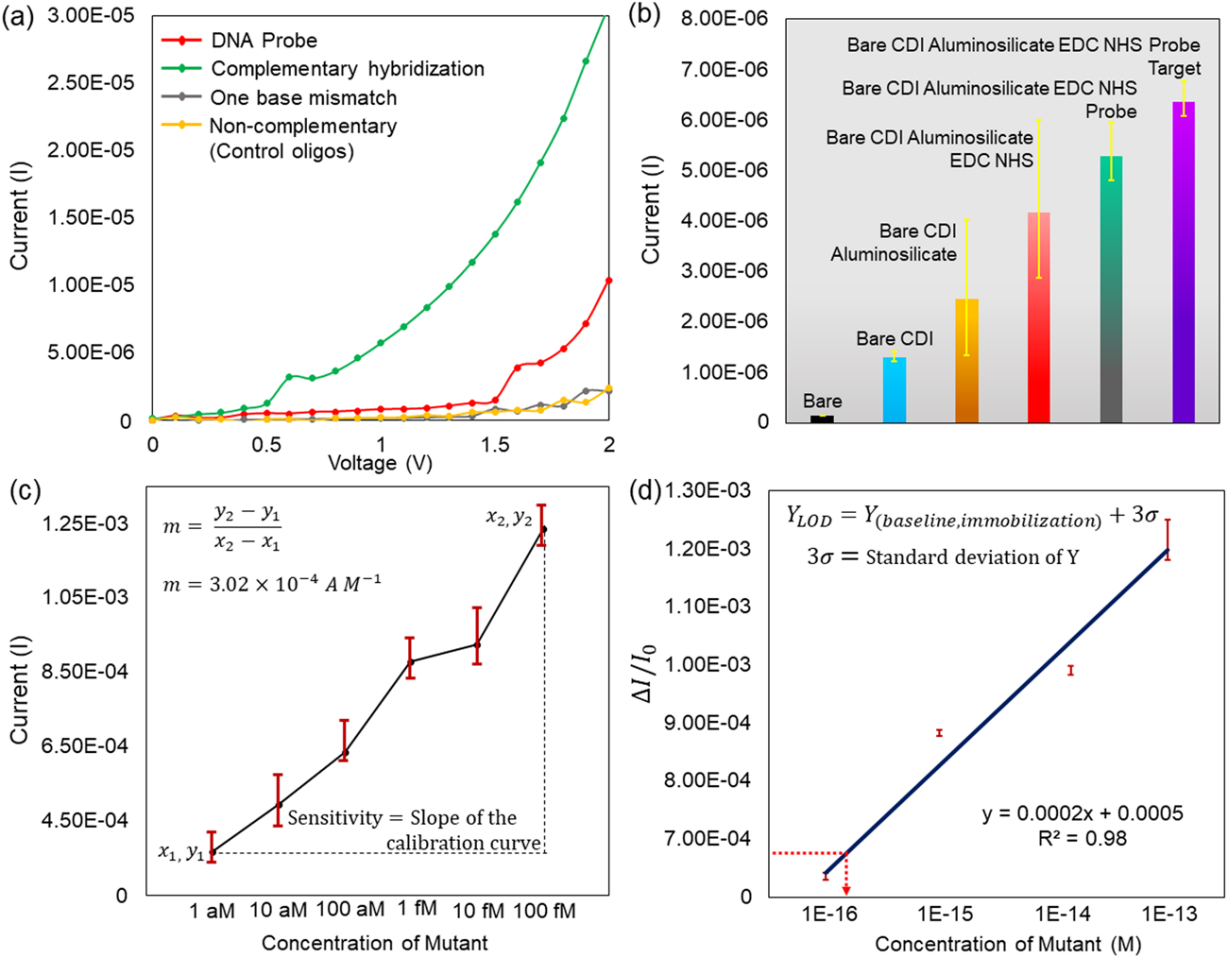
(**a**) Voltammetry signal generated by DNA hybridization. Performed with mutant, wild and non-complementary strands justifying the specificity of genosensor in the detection of EGFR mutation in the presence of single base pair mismatch. (**b**) I-V characteristics generated at every step of functionalization demonstrated the influence of chemical bonding on electron flow through the electrodes. (**c**) The curve represents the voltammetry response amplified by genosensor at different concentration of mutant strand at 2 V. (**d**) Graph plotted shows the relative change of current against the different concentration of mutant at 2 V, revealing the detection limit (LOD).

Figure 6a shows the apparent variation in the voltammetry signal generated by aluminosilicate nanocomposite modified genosensor when tested with mutant (complementary), wild (one base mismatch) and non-complementary targets on the immobilized DNA probe at 100 aM concentration of each target. The prominent curve of complementary hybridization generated due to the high electron shift justifies the significant voltammetry charge variation affected by the specificity of genomic sequences in inducing the genosensor voltammetry signal, evidence the high sensitivity of genosensor. The modification of chemical bonding on the genosensor and the voltammetry signal amplified at 2 V voltage source is illustrated in Fig. 6b. Bare IDE with no chemical modification shows the least current amplified and as the surface chemistry was modified as desired, the current generated by genosensor gradually increased indicating the high electron shift due to the strong chemical bonding and its charge carriers on the genosensor. The sensitivity of genosensor was analyzed using complementary mutant strands from 1 aM to 100 fM concentration against the current amplified by the genosensor. The elevation in the voltammetry signal generated at 2 V as the concentration of mutant strands is increased, it reflects the increasing density of DNA hybridization creating high electron shift through the electrodes. Based on the slope of the calibration curve generated in Fig. 6c, a linear response is observed and high sensitivity of genosensor is computed, which is 3.02E-4 A M^−1^. The sensitivity is compared with a recently reported work. It is found that the genosensor sensitivity is 16 fold higher compared to the proposed electrochemical DNA biosensor in EGFR mutant detection^44^. Therefore, it can be concluded that the proposed genosensor exhibits significant sensitivity due to the excellent physiochemical characteristics of aluminosilicates, further followed by precise surface chemistry in the probe immobilization which creates a huge density of DNA hybridization on genosensor. Further, the limit of detection (LOD) was evaluated to determine the lowest concentration of the mutant strand needed to be immobilized on the genosensor to generate the efficient voltammetry signal indicating EGFR mutation for NSCLC detection. LOD was calculated based on the relative change of current proportional to the logarithm of mutant concentration as plotted in Fig. 6d. The following equation was used to calculate the LOD.

1 $${Y}_{LOD}={Y}_{(baseline,immobilization)}+3\sigma $$

${Y}_{(baseline,immobilization)}$ assumed to be 0 as the background signal generated by genosensor is 0. $3\sigma$ represents the standard deviation of calibration curve regression. LOD of genosensor calculated from the calibration curve is 100 aM of mutant. The obtained LOD promises that aluminosilicate modified genosensor is highly sensitive to detect EGFR mutation at 100 aM mutant concentration which is efficient to detect NSCLC with limited clinical samples. The obtained LOD in the present research is significant in the detection of EGFR mutation in attomolar range compared to recently reported work in literature (Table S1).

## Conclusion

In the research, we have presented the promising EGFR mutation detection for NSCLC using genosensor, where the sensing area enhanced with aluminosilicate nanocomposites. The simple, inexpensive genosensor was successfully fabricated through conventional photolithography technique using aluminium as conductive electrodes. FTIR results presented affirm the precise surface enhancement using aluminosilicate nanocomposites which empower the high-density DNA immobilization on the genosensor. The ∼25 nm size aluminosilicate increases the DNA probe attachment on the genosensor compared to the genosensor with no aluminosilicate, indicated by the voltammetry signal generated during DNA hybridization. The lowest detecting limit of aluminosilicate modified genosensor at 100 aM mutant shows excellent sensitivity of genosensor and its specificity in detection EGFR mutation in the presence of mutant type and single base mismatch. The LOD of aluminosilicate modified genosensor calculated based on $3\sigma$ attained is 100 aM which is extremely low concentration of DNA analyte in the detection of cancerous diseases. The research presented affirms that genosensor enhanced with aluminosilicate nanocomposite is a promising device to detect EGFR mutation at an early stage of NSCLC. The research motivation is justified as the efficient and effortless strategy is successfully developed through aluminosilicate modified genosensor which is highly recommended to diagnose diseases associated with genomic DNA hybridization.

## Supplementary information

Supplementary Information

## Data Availability

Relevant data is available in the Supplementary Source file.
