# Supplementary material for: Aluminosilicate Nanocomposite on Genosensor: A Prospective Voltammetry Platform for Epidermal Growth Factor Receptor Mutant Analysis in Non-small Cell Lung Cancer
Source: Sci Rep. 2019 Nov 19;9:17013. doi: 10.1038/s41598-019-53573-9 (PMC6863915; doi:10.1038/s41598-019-53573-9)
Supplement: Supplementary file 1 — Supplementary Information [file 41598_2019_53573_MOESM1_ESM.pdf]

Supplementary Information

Aluminosilicate Nanocomposite on Genosensor: A Prospective Voltammetry Platform for Epidermal Growth Factor Receptor Mutant Analysis in Non-small Cell Lung Cancer

Santheraleka Ramanathan[1], Subash C.B. Gopinath[1,2*], M.K. Md Arshad[1,3], Prabakaran Poopalan[3], Periasamy Anbu[4], Thangavel Lakshmipriya[1] Farizul Hafiz Kasim[2,5]

[1]Institute of Nano Electronic Engineering, Universiti Malaysia Perlis, 01000 Kangar, Perlis, Malaysia.
[2]School of Bioprocess Engineering, Universiti Malaysia Perlis, 02600 Arau, Perlis, Malaysia.
[3]School of Microelectronic Engineering, Universiti Malaysia Perlis, Pauh Putra, 02600 Arau, Perlis, Malaysia.
[4]Department of Biological Engineering, College of Engineering, Inha University, Incheon 402-751, Republic of Korea.
[5]Centre of Excellence for Biomass Utilization, School of Bioprocess Engineering, Universiti Malaysia Perlis, 02600 Arau, Perlis, Malaysia.

Correspondence to:

Asso. Prof. Dr. Subash C.B. Gopinath
(subash@unimap.edu.my)

Figure S1

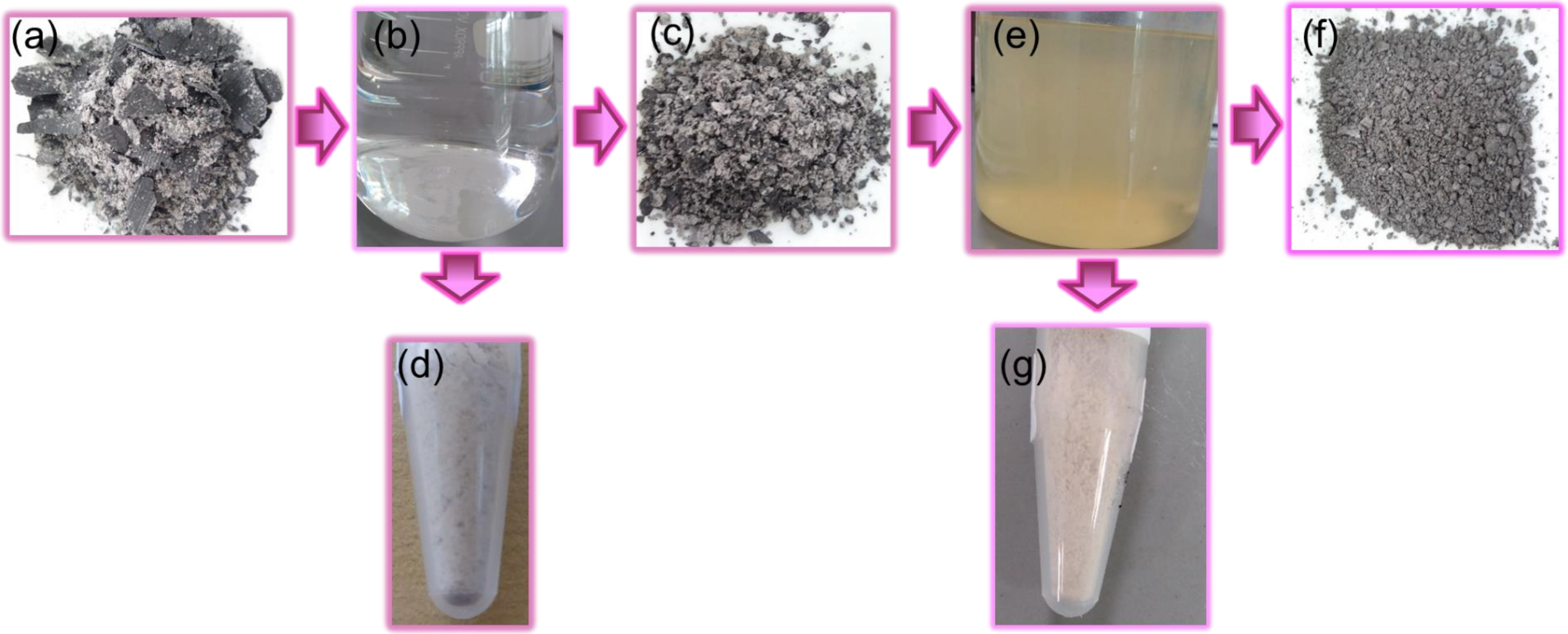

Figure S1: Samples collected at each experimental step during the synthesis of aluminosilicate nanocomposite from joss fly ash. (a) refers to the incinerated joss fly ash, (b) refers to the supernatant of joss fly ash with acidic treatment, (c) refers to ash that undergone acid treatment, (d) refer particles formed after titrated acid leached joss fly ash solution, (e) refers to the supernatant of (c) with alkaline treatment, (f) refers to joss samples that undergone alkaline treatment and (g) refers to aluminosilicate nanocomposite extracted from joss fly ash.

Figure S2

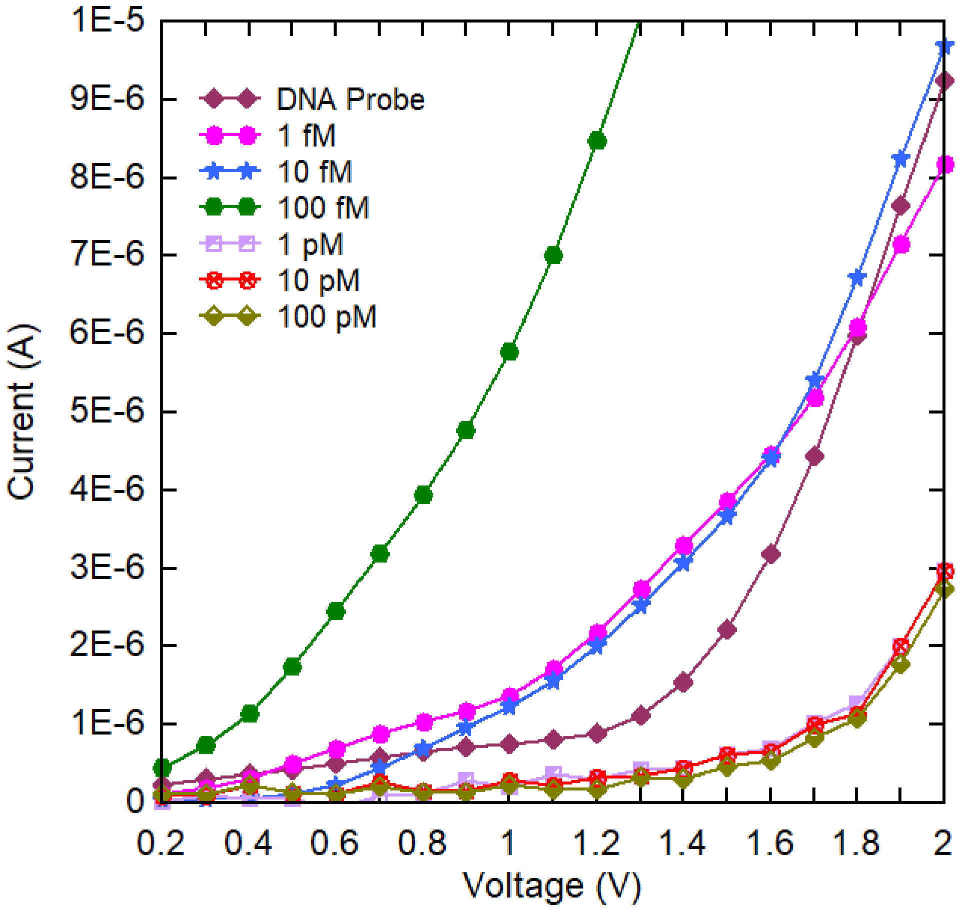

Figure S2: Voltammetry signal amplified by genosensor. The graph shows current-voltage (I-V) characteristics generated by genosensor as the DNA probe was allowed hybridizing with mutant target from 1 fM to 100 pM concentration.

Table S1: Comparison between biosensors established in recent literatures for detection of EGFR mutation.

Device	Detection strategy	Nanomaterial	Limit of Detection	Advantages	References
Electrochemical biosensor	DNA hybridization	Quartz crystal microbalance nanoporous electrode	1 nM	In-situ and high sensitive detection	[1]
Kelvin probe force microscopy	DNA hybridization	Gold nanoparticle	3.3 pM	Label free and high sensitive detection	[2]
Electrochemical biosensor	DNA hybridization	Microfluidic paper based sensing area	0.167 nM	Applicable in clinical diagnosis, good stability and specificity	[3]
Electrochemical biosensor	DNA hybridization	Compromised with oligo-quencher (oligo-Q) and molecular beacon	0.02 ng	Comprehensive, simple and efficient detection	[4]
Electrochemical biosensor	DNA hybridization	Mesoporous carbon nanocomposite and pencil graphite electrode	120 nM	Fast, high selective and sensitive detection	[5]

References

1. Park, H., You, J., Park, C., Jang, K. & Na, S. In-situ and highly sensitive detection of epidermal growth factor receptor mutation using nano-porous quartz crystal microbalance. *Journal of Mechanical Science and Technology* **32**, 1927–1932 (2018).
2. Jang, K., Choia, J., Park, C. & Na, S. Label-free and high-sensitive detection of Kirsten rat sarcoma viral oncogene homolog and epidermal growth factor receptor mutation using Kelvin probe force microscopy. *Biosensors and Bioelectronics* **87**, 222–228 (2017).
3. Tian, T. *et al.* Paper-based biosensor for noninvasive detection of epidermal growth factor receptor mutations in non-small cell lung cancer patients. *Sensors and Actuators, B: Chemical* **251**, 440–445 (2017).
4. Bae, J. H., Jo, S. M. & Kim, H. S. Comprehensive detection of diverse exon 19 deletion mutations of EGFR in lung Cancer by a single probe set. *Biosensors and Bioelectronics* **74**, 849–855 (2015).
5. Shoja, Y., Kermanpur, A. & Karimzadeh, F. Diagnosis of EGFR exon21 L858R point mutation as lung cancer biomarker by electrochemical DNA biosensor based on reduced graphene oxide /functionalized ordered mesoporous carbon/Ni-oxytetracycline metallopolymer nanoparticles modified pencil graphite elec. *Biosensors and Bioelectronics* **113**, 108–115 (2018).
